# Psychometric properties of the ICECAP-SCM capability-wellbeing measure in specialist palliative care units in Austria

**DOI:** 10.1007/s11136-025-04032-8

**Published:** 2025-07-23

**Authors:** Elisabeth Saly, Judit Simon, Karin Brenner, Bert Engelhardt, Gudrun Kreye, Rudolf Likar, Eva Katharina Masel, Veronika Mosich, Mario Molnar, Andrea Passini, Claudia Fischer

**Affiliations:** 1 Department of Health Economics, Center for Public Health, Medical University of Vienna, Vienna, Austria; 2 Palliative Care Ward, Center for Oncology and Hematology, First Department of Medicine, Klinik Ottakring, Vienna, Austria; 3 Palliative Care Ward, Franziskus Spital, Vienna, Austria; 4 Karl Landsteiner University of Health Sciences, Krems, Austria; 5 Division of Palliative Care, Department of Internal Medicine 2, University Hospital Krems, Krems, Austria; 6 Department of Anesthesiology, General Intensive Medicine, Emergency Medicine, Center for Interdisciplinary Pain Therapy and Palliative Medicine, Klinikum Klagenfurt am Wörthersee, Klagenfurt, Austria; 7 Division of Palliative Medicine, Department of Medicine I, Medical University of Vienna, Vienna, Austria; 8 Caritas Socialis CS Hospiz Wien, Vienna, Austria; 9 Palliative Care Ward, Department of Internal Medicine and Haemato-Oncology, LKH Hochsteiermark, Leoben, Austria

**Keywords:** Patient reported outcome measures, Psychometrics, ICECAP-SCM, EQ-5D-5L, IPOS, Palliative care

## Abstract

**Purpose:**

Patient-reported outcome measures (PROMs) with robust psychometric properties are key for healthcare and resource allocation decisions. Palliative Care (PC) presents challenges for PROM assessment including its holistic scope, patients’ poor health and uncertainty about suitable PROMs to capture the value of PC. The ICECAP-SCM capability-wellbeing questionnaire was developed for economic evaluations in PC but its psychometric information is limited. This study assessed the comparative validity of ICECAP-SCM in Austrian specialist PC settings.

**Methods:**

The PallPROMs cohort study collected PROM data for quality-of-life or symptom and concern burden (ICECAP-SCM, EQ-5D-5L, IPOS) alongside clinician ratings at specialist PC units in 12 Austrian hospitals. We assessed the convergent validity and responsiveness based on pre-developed hypotheses, the known-groups validity of ICECAP-SCM and conducted exploratory factor analysis according to COSMIN guidelines.

**Results:**

Of the 293 participating patients, 228 patients had complete PROM data (58% female, 90% cancer-diagnosis). ICECAP-SCM showed ceiling effects (67–85%) in all domains except physical and emotional suffering. As hypothesized, it had moderate correlations with IPOS (*r*=-0.35) and EQ-5D-5L (*r* = 0.35), though the correlation with IPOS was weaker than with EQ-5D-5L. ICECAP-SCM effectively discriminated between patients with different symptom severity levels, and showed responsiveness to improvements. A four-factor structure was identified, with EQ-5D-5L loading on three factors and ICECAP-SCM and IPOS on all four factors.

**Conclusion:**

This study provides evidence of the validity of ICECAP-SCM in specialist PC units. It confirms its ability to provide a broader, more holistic wellbeing information than EQ-5D-5L. However, observed ceiling effects may limit its applicability.

**Supplementary Information:**

The online version contains supplementary material available at 10.1007/s11136-025-04032-8.

## Introduction

Healthcare costs for end-of-life care are substantial [1–3], with evidence indicating that hospital care (e.g., intensive care or emergency room visits) is the primary driver of these expenses [1; 4]. At the same time, approximately one-third of treatments in the last six months of life are non-beneficial. Non-beneficial treatments reflect a mismatch between treatment intensity and expected benefits such as health improvement, survival or quality-of-life enhancements [5]. To mitigate this, Palliative Care (PC), with its holistic approach, can play a key role in reducing unnecessary treatments and improving health-related quality of life (HRQoL) [4, 6].

Specialist PC is provided to patients with particularly complex care needs, and can be delivered through various models, including palliative consultation services, hospices or PC units (PCUs) within hospitals [7]. In specialist PCUs, interprofessional teams deliver care to patients with advanced needs that cannot be addressed by other services, due to medical, nursing, or psychosocial factors [8]. The primary focus is to alleviate symptoms and stabilize patients as much as possible, facilitating their discharge back to their place of residence or transfer to alternative services, such as hospices.

Patient-reported outcome measures (PROMs) are important tools for monitoring health status, assessing HRQoL and wellbeing, and evaluating the impact of interventions and overall care effects [9]. To date, there is no consensus among relevant academic and medical stakeholders and decision-makers on which PROMs are appropriate for assessing effects in PC [10, 11].

The EQ-5D-5L is a generic instrument designed to measure HRQoL and is used to calculate quality-adjusted life years (QALYs). QALYs, a metric that combines quantity and quality of life into a single index, reflect the length of life adjusted by HRQoL based on preferences of the general population [12]. The use of EQ-5D-5L in PC has been debated because PC is holistic in nature and encompasses more than HRQoL, such as spiritual wellbeing and patient dignity [13–15].

In recent years, PROMs tailored to the PC setting, such as the Integrated Palliative care Outcome Scale (IPOS) have been developed [16]. However, the IPOS lacks a preference-based value set and cannot generate QALYs, which are the currently preferred outcome for economic evaluations in health technology assessments.

To capture broader aspects of wellbeing beyond HRQoL in economic evaluations, the ICECAP tools have been developed [17]. These are grounded in Sen’s capability approach and define wellbeing in terms of the extent to which a person is capable of achieving a personally valuable life [18]. Interest in this approach for economic evaluations has grown, with various instruments now available [19]. For the palliative and end-of-life care context, the ICECAP Supportive Care Measure (ICECAP-SCM) was developed and published in 2013 for use in economic evaluations [20]. The English ICECAP-SCM has been tested for construct validity and responsiveness in UK hospice PC settings. Additionally, its feasibility for completion has been demonstrated among patients with end-stage organ failure receiving hospital care [21, 22]. In 2021, Gühne and colleagues translated the English ICECAP-SCM into German and subsequently assessed its content validity [23]. However, the psychometric properties of both the German and English ICECAP-SCM have not been tested in settings outside of these initial contexts. Given the diversity of PC settings and patient profiles [24], assessing the ICECAP-SCM’s psychometric properties in specialist PCUs for patients with the most severe symptomatology across different diseases is highly relevant.

The aim of this study was to comprehensively assess the psychometric properties of the ICECAP-SCM, including convergent validity, known-groups validity, responsiveness and structural validity, in relation to the EQ-5D-5L and the IPOS in specialist PCUs in Austrian hospitals.

## Methods

We followed the COSMIN (Consensus-based Standards for the selection of health status Measurement Instruments) reporting guideline for studies on measurement properties of PROMs [25] Supplementry material S1.

### Population and data collection

We used data from the PallPROMS study, a national multicenter cohort study that collected paper-based PROMs from patients with PC needs in specialist PCUs across 12 Austrian hospitals between October 2021 and April 2023 [26]. Participants were aged 18 years or older, able to understand German or English, and admitted to a participating PCU. Patients were excluded if they were incapable of completing a questionnaire, even with assistance, or if they were unwell or distressed, as judged by the treating health care professional. All patients provided informed consent. At baseline, healthcare professionals collected data on patients’ socio-demographics and clinical information. After one week, a follow-up assessment took place. The one-week interval was chosen due to the potential for rapid health deterioration and to increase the inclusion of more patients. Healthcare professionals reported whether patients completed the PROMs independently or with assistance from healthcare professionals or relatives/friends. Assistance refers to basic help with writing or reading, as well as interview-like assessments. Ethics approval for the PallPROMS study was obtained (see *Ethics approval*).

### Instruments

In our study, we examined the PC-specific ICECAP-SCM in comparison to the PC-specific IPOS and generic EQ-5D-5L (including EQ-VAS).

#### ICECAP-SCM

The ICECAP-SCM is a capability-based measure developed for assessing (palliative) care at the end of life. It consists of seven items (*choice*, *love and affection*, *physical suffering*, *emotional suffering*, *dignity*, *being supported*,* preparation)*, scored on a four-level scale, where 4 represents the highest level of capability and 1 the lowest. We used the available tariffs for Germany, with scores ranging from 0 (no capability in all attributes) to 1 (full capability in all attributes). The tariffs based on combined data (including discrete-choice-experiment and best-worst-scaling valuation data) were used for this study to create the ICECAP-SCM index value [27]. The ICECAP-SCM sum score ranges from 7 (worst) to 28 (best).

#### EQ-5D-5L

The EQ-5D-instruments, developed by the EuroQol Group, are widely used, self-reported, generic measures of HRQoL, with established validity and reliability across different health conditions and populations [28]. The EQ-5D-5L includes five items: *mobility*, *self-care*, *usual activities*, *pain/discomfort* and *anxiety/depression*, scored on a 5-point Likert scale from 1 (best) to 5 (worst). The EQ-VAS is a vertical visual analogue scale where patients rate their health from 0 (worst imaginable health) to 100 (best imaginable health). In the absence of a country-specific value set, the EQ-5D manual recommends using one from a country or population with similar characteristics [29]. As no Austrian EQ-5D-5L value set exists, we used the German value set due to cultural and linguistic similarities. This value set produces index values ranging from − 0.661 to 1, where 0 represents death and 1 perfect health [30].

#### IPOS

The IPOS combines items from the Palliative Care Outcome Scale (POS) [31] and its symptom list (POS-S) into one integrated measure [16]. We used the 7-day-version for patient self-report, which includes 20 items: a free-text question on main concerns, 17 items on physical, social, psychological and spiritual needs (scored on a 5-point Likert scale from 0 (best) to 4 (worst)), another free-text item asking for additional symptoms to be specified and rated, and one item to report who completed the measure (patient alone, with family help, with staff help). The total score is the sum of the 17 standardized questions and ranges from 0 to 68 [16; 32]. Higher scores indicate greater symptom burden and therefore poorer outcomes. The IPOS has been tested for its validity, reliability and responsiveness in different settings in the UK and Germany [32], and validated in various languages, cultures and health conditions but not in Austria [33]. Testing PROMs in the local context ensures that tools accurately reflect the population’s health perceptions, values and experiences, providing reliable and contextually relevant outcomes [34].

#### Clinician-reported health assessment of the patients

At follow-up assessment, clinicians assessed and recorded changes in the patient’s overall health condition based on their clinical judgement. They categorized the patient’s general health state as either no change, improved or deteriorated since the previous assessment.

### Analyses

#### Construct validity

We tested the convergent validity of the measures by exploring the correlation between the baseline ICECAP-SCM, EQ-5D-5L, EQ-VAS and IPOS scores. Prior to conducting the analyses, hypotheses about expected relationships between the scores were developed and discussed by the study team (Table 1). Correlations were interpreted as strong (≥ 0.50/≤-0.50), moderate (0.30–0.49/-0.30 to -0.49), weak (0.10–0.29/-0.10 to -0.29) and none (-0.10 to 0.10), following Cohen’s guidelines [35].

**Table 1:** Initial hypotheses about correlations between the ICECAP-SCM, EQ-5D-5L, EQ-VAS and IPOS scores

Hypotheses	
H1	There will be an at least moderate negative correlation (*r* ≤ -0.30) between the ICECAP-SCM and the IPOS, as they are both directed at patients in palliative/EOL care.
H2	The correlation between the ICECAP-SCM and the IPOS is expected to be stronger than the correlation between the ICECAP-SCM and the EQ-5D-5L because both the ICECAP-SCM and the IPOS focus on concerns specific to palliative and end-of-life care.
H3	There will be a moderate correlation (*r* = 0.30–0.49) between the ICECAP-SCM and the EQ-5D-5L since both measure quality of life, but their constructs differ.
H4	There will be a weak correlation (*r* = 0.10–0.29) between the ICECAP-SCM and the EQ-VAS since the EQ-VAS is a generic, non-preference-based measure.
H5	The correlation between the IPOS and the EQ-5D-5L is expected to be negative and moderate (*r* = -0.30 to -0.49) since they are both focusing on health-related aspects.

Note: EQ-VAS = Visual analogue scale, ICECAP-SCM = ICECAP-Supportive Care Measure, IPOS = Integrated Palliative care Outcome Scale; Correlations between IPOS and the other instruments are expected to be negative as the scores indicating better health are reverse

To assess the known-groups validity of the ICECAP-SCM, namely its ability to differentiate between patient groups [36], we conducted a comparative analysis of the ICECAP-SCM index values across different patient groups according to symptom severity [37]. These were defined by the Karnofsky Performance Status (KPS) [38], pain score (assessed by both, the patient and the clinician, based on the Numeric Rating Scale (NRS)) and IPOS symptom score (details are presented in Supplementry material S2). We conducted one-way ANOVA and linear regression analyses, adjusted for age and sex using ICECAP-SCM index values as dependent variable, and KPS, pain (patient and clinician) and IPOS symptom score as categorical independent variables, setting the most severe group as the reference.

#### Responsiveness

We evaluated the responsiveness of the ICECAP-SCM, which refers to its ability to detect changes in the measured construct over time in three ways [36].

1. Anchor-based approach: We used the clinician’s assessment of changes in the patient’s health (whether it deteriorated, improved or remained unchanged) as an anchor. This external indicator helped identify changes unlikely due to chance [39].
2. Distribution-based approach: We categorized individuals based on their changes in PROM scores. Given the limited data on minimally important differences for patients in PC settings, we defined a change of half a standard deviation (SD) of the baseline EQ-5D-5L index, EQ-VAS and IPOS scores as meaningful. Patients were classified as “improved”, “worsened” or “unchanged”. Changes in ICECAP-SCM scores were then calculated for each group, and differences across the three groups were analyzed using one-way ANOVA and post-hoc analyses. The magnitude of change was classified as small (< 0.50), moderate (0.50–0.79) or large (≥ 0.80) using the standardized response mean (SRM) [40], calculated as the ratio of the mean change between baseline and follow-up scores to the SD of the change scores [41]. Additionally, we examined the agreement of the proportion of participants who improved, deteriorated or remained unchanged, according to the ICECAP-SCM, other PROMs and clinician assessment.
3. Construct approach: This approach was based on hypothesis testing, similar to construct validity. The details of this analysis are described in Appendix A3.

#### Exploratory factor analysis

We conducted an exploratory factor analysis (EFA) to determine whether the items of the ICECAP-SCM, IPOS and EQ-5D-5L share a common set of underlying factors or measure distinct constructs. Prior to conducting the EFA, we evaluated the suitability of the data using the Kaiser-Meyer Olkin measure and the Bartletts test for sphericity [42]. The number of factors were based on the Kaiser Criterion and the scree plot. Polychoric correlations were used, as they are suitable for ordinal data. We used orthogonal oblimin rotations to rotate the factors. The minimum loading criterion was 0.32 and factor loadings were considered poor (0.32–0.44), fair (0.45–0.54), good (0.55–0.62), very good (0.63–0.70) or excellent (> 0.71) [43].

Due to the large number of missing values in the IPOS, a sensitivity analysis was conducted using a larger sample. Imputed values for symptoms were determined based on clinical expert feedback. Missing values for IPOS symptoms were imputed as “Not at all”, assuming that blank responses likely indicated irrelevance. Since the sensitivity analysis produced results highly consistent with the primary analysis, we report only the results of the primary analysis.

In all analyses, a *P*-value of less than 0.05 was considered statistically significant. In the primary analysis, no imputation of missing values was performed. All the primary analyses were conducted on observations with complete data on all assessed PROMs (ICECAP-SCM, EQ-5D-5L, EQ-VAS, IPOS) using Stata 18.0.

## Results

### Patient characteristics

Of 2289 potential patients admitted to the participating specialist PCUs, 293 participated in the baseline assessment. Among them, 277 patients fully completed the ICECAP-SCM, 275 the EQ-5D-5L, 273 the EQ-VAS and 231 the IPOS. Overall, 228 patients completed all PROMs and were included in the analyses. At one-week follow-up, 148 patients completed all PROMs. More information on the feasibility of PROM assessment in specialist PCUs is reported elsewhere [26]. The time between baseline and follow-up ranged from 2 to 18 days, (mean/median: 7 days, SD: 2.17 days). Patient characteristics are presented in Table 2, mean age was 70 years (range: 23–96) and 58% were female. Almost half the participants were married, over 60% were Christians, educational levels were commonly lower than A-levels (69%), and 90% had cancer as their primary diagnosis.

**Table 2:** Sample characteristics at baseline

	*N*	Mean (SD) or %	Min - Max
Sample size	228		
Mean age at admission	196	70.3	23.0–96.0
Missing	32	14%	
Sex			
Female	132	58%	
Male	94	41%	
Missing	2	1%	
Marital status			
Single	29	13%	
Married	104	46%	
Divorced	52	23%	
Widowed	38	17%	
Missing	5	2%	
Educational level			
Lower than A-levels	157	69%	
A-levels	21	9%	
Higher than A-levels	34	15%	
Missing	16	7%	
Religion			
Christian	139	61%	
Unaffiliated	58	25%	
Other religions	3	1%	
Not reported	20	9%	
Missing	8	4%	
Primary diagnosis			
Cancer	206	90%	
Non-cancer	9	4%	
Missing	13	6%	
ICECAP-SCM	228	0.8 (0.1)	0.2–1.0
EQ-5D-5L	228	0.4 (0.3)	-0.6–1.0
IPOS	228	30.0 (9.7)	5.0–54.0
EQ-VAS	228	43.7 (22.0)	0.0–100.0

Note: EQ-VAS = Visual analogue scale, ICECAP-SCM = ICECAP-Supportive Care Measure, IPOS = Integrated Palliative care Outcome Scale, Max = Maximum, Min = Minimum, *N* = frequency, SD = Standard deviation

### Analyses

#### Description of the sample

**Fig. 1:**
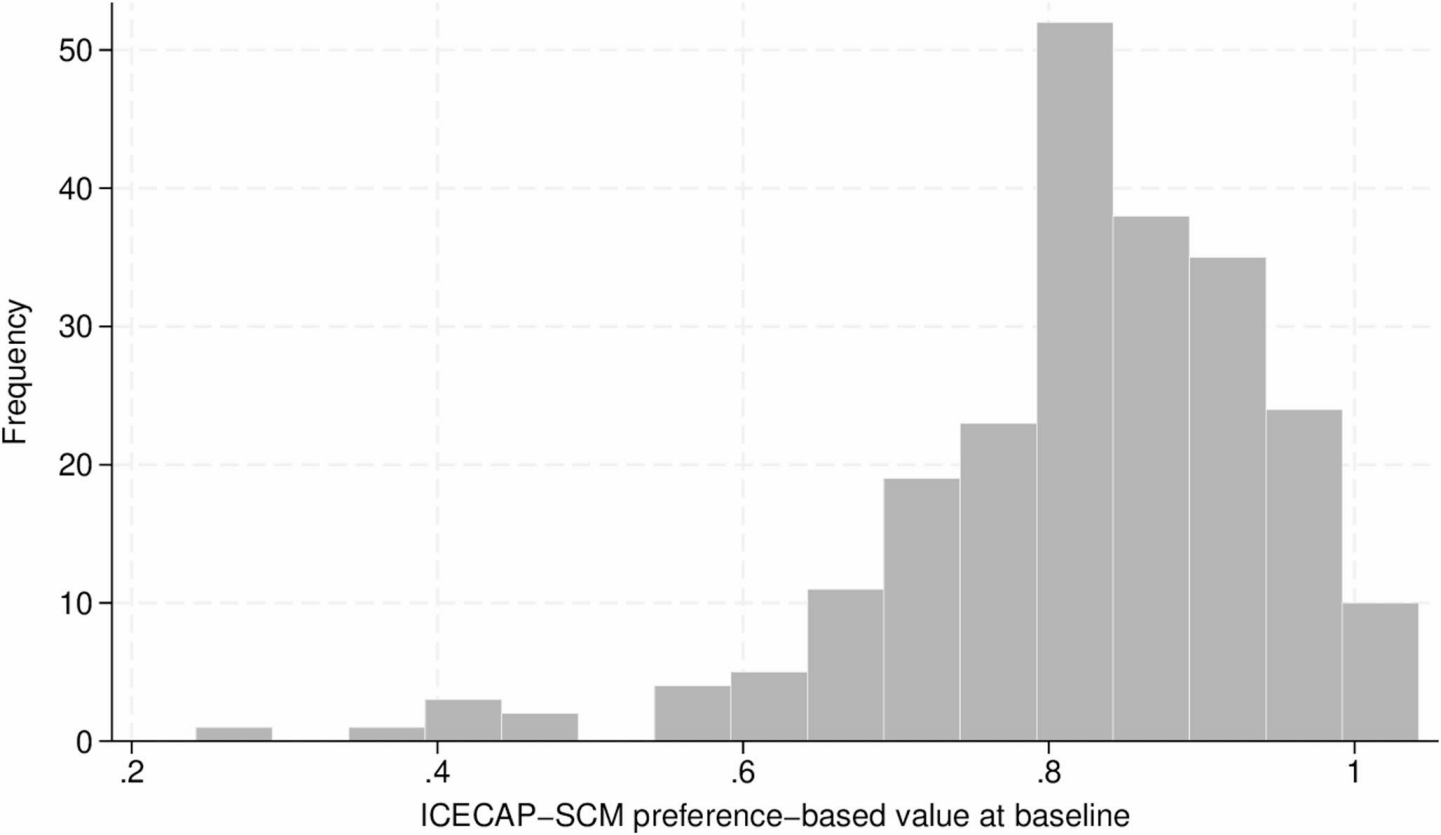
Histogram of ICECAP-SCM index values (0–1) at baseline (*n* = 228)

The distribution of the baseline ICECAP-SCM index values were left-skewed, while the ICECAP-SCM sum scores exhibited a distribution closer to normal (Figs. 1 and Appendix A2- Fig. 3). Mean ICECAP-SCM sum score was 23.5 and ICECAP-SCM index was 0.8, with ten patients (4.4%) reporting full capability. Ceiling effects were present in five ICECAP-SCM domains, as most patients indicated the highest level of capability in *choice* (79%), *love and affection* (76%), *dignity* (85%), *being supported* (84%) and *preparation* (67%). Mean EQ-5D-5L index value was 0.4, with two patients (0.9%) reporting perfect health and 33 (14.5%) a health status worse than death. The EQ-VAS ranged from 0 to 100, with 75% of participants rating their health status 50 or lower. The IPOS ranged from 5 to 54 with a mean of 30 and a fairly normal distribution.

#### Convergent validity

Table 3 presents the correlations between the PROM scores. The correlations between the ICECAP-SCM and the EQ-5D-5L (*r* = 0.35) and IPOS (*r*=-0.35) were lower than between the EQ-5D-5L and IPOS (*r*=-0.43). Overall, the correlations with the EQ-VAS were the lowest (*r* = 0.16 to -0.31). Of the five hypotheses tested, data supported four (H1,H3,H4,H5), whereas H2 was not supported, as the ICECAP-SCM showed a similar correlation with EQ-5D-5L and IPOS, rather than a stronger correlation with IPOS.

**Table 3:** Spearman rank correlation coefficients of ICECAP-SCM, EQ-5D-5L, EQ-VAS and IPOS baseline scores (*n* = 228)

	ICECAP-SCM	EQ-5D-5L	EQ-VAS	IPOS
ICECAP-SCM	1	0.35**	0.16*	-0.35**
EQ-5D-5L		1	0.27**	-0.43**
EQ-VAS			1	-0.31**
IPOS				1

Note: EQ-VAS = Visual analogue scale, ICECAP-SCM = ICECAP-Supportive Care Measure, IPOS = Integrated Palliative care Outcome Scale; **P < 0.001, *P < 0.05; Correlations were interpreted per Cohen’s guidelines, strong (≥ 0.5) (in bold), moderate (0.30–0.49) (in italic), weak (0.10–0.29); ICECAP-SCM and EQ-5D-5L refer to the index values and IPOS to the sum scores

#### Known-groups validity

The ANOVA results revealed significant differences in ICECAP-SCM index values between groups based on the KPS (*P* = 0.005), patient-assessed pain (*P* = 0.0001) and IPOS symptom burden (*P* = 0.013). No significant differences were observed between groups based on clinician-assessed pain (*P* = 0.133). As shown in Table 4, the ICECAP-SCM index values were consistently higher for the less severe patients across all measures. In the univariable analysis, the ICECAP-SCM showed statistically significant differences across groups based on KPS, patient-assessed and clinician-assessed pain scores as well as IPOS symptom burden. After adjusting for age and sex, significant associations remained for KPS, patient-assessed pain scores and the IPOS symptom burden.

**Table 4:** Unadjusted and adjusted associations between the ICECAP-SCM and symptom severity

		ICECAP-SCM			Univariable analysis		Adjusted for age and sex	
		*n*	mean	SD	coef.	*P*	coef.	*P*
KPS (*n* = 228)	Low performance*	65	0.78	0.12				
	Moderate performance	106	0.83	0.12	0.05	**0.004**	0.06	**0.001**
	High performance	57	0.84	0.12	0.06	**0.004**	0.06	**0.009**
Pain (NRS) patient (*n* = 220)	Severe Pain*	25	0.72	0.17				
	Moderate pain	55	0.81	0.12	0.09	**0.002**	0.08	**0.023**
	Mild pain	64	0.83	0.12	0.10	**< 0.001**	0.08	**0.049**
	No pain	76	0.85	0.10	0.13	**< 0.001**	0.12	**0.002**
Pain (NRS) clinician (*n* = 214)	Severe Pain*	14	0.76	0.16				
	Moderate pain	46	0.81	0.11	0.06	0.129	0.06	0.224
	Mild pain	85	0.82	0.13	0.06	0.081	0.05	0.354
	No pain	69	0.84	0.11	0.08	**0.022**	0.09	0.075
IPOS Symptom burden (*n* = 228)	Severe*	81	0.80	0.11				
	Moderate	113	0.82	0.13	0.02	0.299	0.02	0.390
	Low	34	0.87	0.12	0.07	**0.003**	0.06	**0.014**

Note: coef. = coefficient, ICECAP-SCM = ICECAP-Supportive Care Measure, IPOS = Integrated Palliative care Outcome Scale, KPS = Karnofsky Performance Status, NRS = Numeric Rating Scale, * indicates the reference group for comparison, *P* = *P* value, SD = Standard deviation; bold indicates significant results

#### Responsiveness

**Fig. 2:**
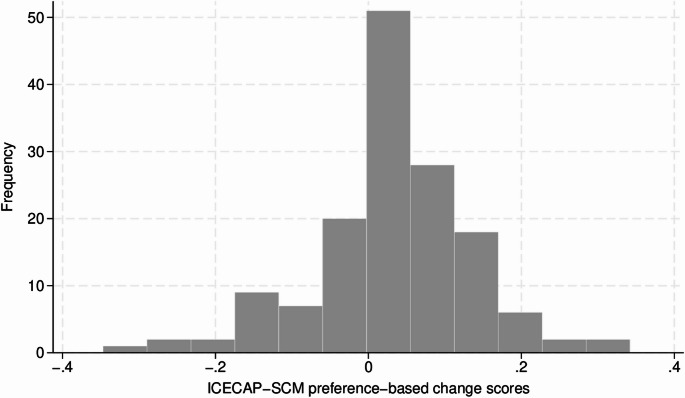
ICECAP-SCM change scores from baseline to one-week follow-up assessment

Figure 2 shows the distribution of the ICECAP-SCM change scores from baseline to follow-up assessment. The mean change of the 148 complete cases from baseline to follow-up assessment was 0.03 (ICECAP-SCM), 0.06 (EQ-5D-5L), 7.07 (EQ-VAS) and − 4.99 (IPOS), indicating an overall improvement.

For the anchor- and distribution-based approach, Table 5 shows that the mean change of the ICECAP-SCM index aligned with the changes indicated by other PROMs, also compared to the external anchor clinician assessment. The ANOVA results indicated statistically significant differences in ICECAP-SCM change scores across the three groups as per EQ-5D-5L index and IPOS, but not clinician assessment and EQ-VAS. The post-hoc test results are presented in Appendix A3 - Table 7. Most of the SRMs were small, except for the improved group according to EQ-5D-5L, which showed a moderate effect. The agreement between changes in ICECAP-SCM index scores and the anchor instruments is detailed in Appendix A3 - Table 8. To summarize, agreement was highest with the IPOS (43–64%), followed by the EQ-5D-5L (25–60%). The construct approach showed that correlations between the change scores of ICECAP-SCM and EQ-5D-5L as well as IPOS were weak, contrary to expectations. Details are presented in Appendix A3– Tables 8 and 9.

**Table 5:** Responsiveness of the ICECAP-SCM to changes in health status

Instrument (no. of complete cases)	Change in instrument scores^1^	*n*	ICECAP-SCM				
			Mean (SD) baseline	Mean (SD) follow-up	Mean change (SD)	*P* ^2^	SRM^3^
Clinician’s assessment (*n* = 144)	Improved	80	0.82 (0.13)	0.86 (0.12)	0.04 (0.10)	0.069	n/a
	Worsened	23	0.83 (0.13)	0.82 (0.17)	-0.02 (0.14)		n/a
	No change	41	0.83 (0.11)	0.86 (0.12)	0.03 (0.09)		n/a
EQ-5D-5L (*n* = 148)	Improved	50	0.80 (0.14)	0.85 (0.13)	0.06 (0.08)	**0.014**	*0.65*
	Worsened	28	0.83 (0.11)	0.82 (0.18)	-0.02 (0.14)		-0.12
	No change	70	0.83 (0.13)	0.86 (0.10)	0.03 (0.10)		0.30
EQ-VAS (*n* = 148)	Improved	48	0.83 (0.14)	0.88 (0.13)	0.04 (0.10)	0.254	0.43
	Worsened	25	0.80 (0.12)	0.80 (0.17)	0.00 (0.12)		-0.25
	No change	75	0.82 (0.13)	0.85 (0.11)	0.03 (0.10)		0.17
IPOS (*n* = 148)	Improved	74	0.81 (0.13)	0.87 (0.11)	0.06 (0.09)	**< 0.001**	0.44
	Worsened	16	0.82 (0.15)	0.78 (0.18)	-0.04 (0.17)		-0.34
	No change	58	0.83 (0.12)	0.84 (0.13)	0.01 (0.09)		0.11

Note: EQ-VAS = Visual analogue scale, ICECAP-SCM = ICECAP-Supportive Care Measure, IPOS = Integrated Palliative care Outcome Scale, SD = Standard deviation; ^1^The categorization of the changes in EQ-5D-5L, EQ-VAS and IPOS scores between baseline and 1-week follow-up in improved, worsened or no change was based on a change of 0.5 standard deviation of the mean baseline score; ^2^*P* values are from one-way ANOVA tests; ^3^SRM = Standardized response mean = (Mean follow-up score– Mean baseline score) / SD of baseline score; SRM values < 0.5 small, 0.5–0.79 moderate (indicated in italic), >= 0.8 large

#### Exploratory factor analysis

The Kaiser-Meyer-Olkin measure was 0.772 for the ICECAP-SCM, IPOS and EQ-5D-5L items, and the Bartlett’s test was significant (*P* < 0.001), indicating suitability for factor analysis. Following the analysis of the scree plot of Eigenvalues and the Kaiser criterion (Appendix A4 - Fig. 4), we applied a four-factor model.

Table 6 shows the factor loadings of the ICECAP-SCM and IPOS domains across all four factors and EQ-5D-5L domains across three factors. Factor 1, reflecting *emotional distress and wellbeing*, includes one domain from ICECAP-SCM, one from EQ-5D-5L domain and seven from IPOS. Factor 2, addressing *mobility and physical functioning*, reflects one ICECAP-SCM domain, three EQ-5D-5L domains and four IPOS domains. Factor 3, related to *emotional and practical support*, reflects most ICECAP-SCM domains (*n* = 5) and three IPOS domains (s*haring feelings*, *being informed* and *practical problems being addressed*). Factor 4 is associated with *pain and discomfort*. Factor loadings were strong (> 0.55) for 18 domains, but weak (< 0.45) for eight domains (e.g. several IPOS symptoms). Three domains exhibited cross-loadings (e.g. ICECAP-SCM *Physical suffering* loading on factor 2 and 4). Additionally, seven domains demonstrated high uniqueness (> 0.70) (e.g. IPOS *practical problems being addressed*).

**Table 6:** Rotated factor loadings (*n* = 228)

Variable	Factor 1: Emotional distress and wellbeing	Factor 2: Mobility and physical functioning	Factor 3: Emotional and practical support	Factor 4: Pain and discomfort	U
ICECAP-SCM					
Choice			0.65		0.56
Love and Affection			0.60		0.61
Physical suffering		-0.37		-0.46	0.58
Emotional suffering	-0.70				0.46
Dignity			0.64		0.59
Being supported			0.63		0.60
Preparation			0.50		0.73
EQ-5D-5L					
Mobility		0.84			0.28
Selfcare		0.83			0.30
Usual activities		0.81			0.33
Pain/discomfort				0.68	0.47
Anxiety	0.65				0.53
IPOS					
Symptom 1: Pain				0.74	0.41
Symptom 2: Shortness of breath	0.40				0.81
Symptom 3: Weakness/lack of energy	0.39	0.38			0.60
Symptom 4: Nausea				0.69	0.43
Symptom 5: Vomiting				0.68	0.51
Symptom 6: Poor appetite				0.46	0.60
Symptom 7: Constipation				0.38	0.79
Symptom 8: Sore or dry mouth		0.32			0.75
Symptom 9: Drowsiness	0.34	0.35			0.68
Symptom 10: Poor mobility		0.81			0.32
Anxiety patient	0.75				0.44
Anxiety family	0.46				0.75
Depression	0.81				0.34
Feeling at peace	0.71				0.45
Sharing feelings			-0.59		0.55
Being informed			-0.32		0.79
Practical problems being addressed			-0.38		0.82

Note: ICECAP-SCM = ICECAP-Supportive Care Measure, IPOS = Integrated Palliative care Outcome Scale, U = Uniqueness; Factor loadings < 0.32 are blank, factor loadings strength: poor (0.32–0.44), fair (0.45–0.54), good (0.55–0.62), very good (0.63–0.70) excellent (> 0.71)

## Discussion

Our study aimed to evaluate the psychometric properties of the ICECAP-SCM in specialist PCUs in Austrian hospitals including convergent and known-groups validities, responsiveness and shared underlying factors with the EQ-5D-5L and IPOS.

### Comparison of the values to other reference groups

The mean scores were 23 for the ICECAP-SCM sum and 0.8 for the ICECAP-SCM index, 0.4 for the EQ-5D-5L index, 44 for the EQ-VAS and 30 for the IPOS. Our results align generally with those of previous studies in different settings and countries. The ICECAP-SCM sum score in our sample was similar to that of a Dutch group of older adults (≥ 70 years) with frailty and complex care needs [44]. In contrast, the ICECAP-SCM index in our sample was higher than that of a UK hospice population [21] suggesting variations related to the different settings or tariffs used. The EQ-5D-5L index was comparable to scores in a UK hospice population [21] and in UK older patients (≥ 75 years) with a poor prognosis [45] but lower than of a German cohort receiving specialist palliative home care [46], implying that patients in hospices or hospitals feel worse than those cared for at home. The EQ-VAS was similar to both, the German [46] and UK cohorts [45]. As expected, the EQ-5D-5L index and EQ-VAS in our study were lower than the reference values for the German general elderly population [47]. Finally, the IPOS in our sample was similar to the UK cohort [45] but higher than a Dutch group of older adults [44].

In this study, two patients reported perfect health on the EQ-5D-5L, suggesting that some patients either misunderstood the question, adapted their response to their situation, or found the domains irrelevant. The observed ceiling effects across five ICECAP-SCM domains, could indicate limitations of the ICECAP-SCM in the specialist PCU setting. The ICECAP-SCM’s current four-level response options, not permitting a response of *No problems at all*, may not fully capture patients’ experiences and subjective adaptation mechanisms in the specialist PCU context. A five-level-version might be more appropriate for this setting, contrary to earlier valuation work [48]. Another possible explanation for these ceiling effects is that these ICECAP-SCM domains may not be relevant to the specialist PCU setting, or that the studied population may have adapted to their situation. These ceiling effects limit the measure’s ability to detect differences or changes over time.

The findings showed high capability ratings despite poor health status, highlighting the importance of considering adaptive preferences and response shift mechanisms [49]. People receiving PC often experience significant health deterioration, which can lead to adjustments in their expectations and perceptions of their health status [50]. This adaptation may cause them to undervalue potential health improvements, thereby skewing self-reported measures and creating a discrepancy between actual health status and perceived capabilities. Such undervaluation can potentially disadvantage this population in funding decisions [51]. Understanding these mechanisms is important for accurate interpretation of health status and equitable funding in PC.

### Construct validity

Our findings indicate that the ICECAP-SCM and the EQ-5D-5L measure different constructs, whereas the EQ-5D-5L and the IPOS assess more closely related constructs. These results align with earlier studies. One study evaluated convergent validity in UK hospice settings, and found a strong correlation between the ICECAP-SCM and the POS-S, and a moderate correlation between the ICECAP-SCM and the EQ-5D-5L [21]. In our study, the correlation between the ICECAP-SCM and the IPOS was moderate, similar to findings from a Dutch sample of older people with frailty and complex care needs [44]. However, the correlation between the EQ-5D-5L and the EQ-VAS was lower compared to studies conducted in other countries and patient groups [52–54]. For example, a responsiveness study in patients with stroke reported a moderate correlation, in contrast to the weak correlation observed in our study [52]. This suggests that patients in specialist PC settings may adapt their perception of optimal health to align with their disease [51], or they may focus more on their current feelings rather than their overall health. Generally, the recall periods of the ICECAP-SCM, EQ-5D-5L (day of assessment) and the IPOS (one week) differ, which could lead to potential discrepancies in responses to similar items across the measures.

The ICECAP-SCM demonstrated the ability to discriminate between patients with varying levels of severity, as measured by the KPS, IPOS symptom burden, patient-reported and clinician-reported pain. The strength of its ability to differentiate between known-groups varied between the patient-reported and clinician-reported pain, indicating that pain is assessed differently depending on the perspective. To our knowledge, this is the first study to assess the known-groups validity of the ICECAP-SCM.

### Responsiveness

In our study, the mean change between the ICECAP-SCM scores at baseline and after one week was 0.03 (SD 0.10). Comparable levels of change have been reported by Myring and colleagues in hospice settings, with a mean change of -0.01 (SD 0.14) after four weeks [21]. This suggests that the ICECAP-SCM is respondent to change, although at small magnitude. Key questions remain regarding how much change can be expected within a week and how likely this specific patient population is to experience persistent change. Although deterioration is expected due to the natural progression of the disease and the patients’ poor health status, improvements may occur as a result of receiving specialist PC designed to alleviate symptoms. It is important to note that domains such as dignity and preparation may require more than one week to exhibit change, or may even necessitate a change in the care setting. Consequently, the follow-up timeframe of one week is likely insufficient to detect meaningful changes in these domains, as such changes may take longer to become apparent. Nevertheless, the ICECAP-SCM change scores generally aligned with other measures, particularly the IPOS, indicating valid responsiveness as the IPOS is a well-validated measure in different PC settings [33]. While the ICECAP-SCM change scores matched the clinicians’ assessments of patient’s health changes in most cases, the remaining inconsistencies suggest that the ICECAP-SCM captures aspects of change not prioritized by clinicians.

### Exploratory factor analysis

The EFA revealed four underlying factors shared by the PROMs of interest: *Emotional distress and wellbeing*, *Mobility and physical functioning*,* Emotional and practical support*, and *Pain and discomfort*. The ICECAP-SCM loaded strongly onto the *emotional and practical support* factor, shared with the IPOS domains *sharing feelings*,* being informed* and *practical problems being addressed*. The EQ-5D-5L loaded strongly onto the *mobility and physical functioning*, as did some IPOS symptoms, but to a weaker extent. All three instruments contributed to the factors *emotional distress and wellbeing*, as well as *pain and discomfort*. These findings indicate both redundant and complementary domains across the measures. Among the three PROMs examined, the IPOS appears to be the most comprehensive one. However, it is important to note that some domains exhibited high uniqueness, suggesting that their variances are not well explained by the factor structure. Additionally, some factor loadings were weak and cross-loadings were present, limiting the reliability of the factor structure. Our sample size of 228 participants does not achieve the often recommended subject-to-item ratio of 10:1 but meets a ratio of 7:1 and exceeds a sample size of 100, both considered good quality criteria for factor analyses by Terwee et al. [55]. Nonetheless, the relatively small sample size may affect the reliability and generalizability of the factor structure. Therefore, we recommend that future studies with larger samples replicate our findings to further validate the factor structure.

### Implications for research and practice

A study in Australian PC settings with high completion rates of PROMs suggests that comprehensive tools focusing on HRQoL at key time points are more effective than frequently administered shorter tools [56]. According to our EFA findings, the IPOS appears promising as it covers all identified underlying factors. However, it is not suitable for QALY development. Efforts have been made to adapt one of the POS-family instruments for use in economic evaluations, the POS-E, but further steps like obtaining preference weights have yet to be realized [57]. Future research should focus on how measures like the IPOS can be mapped onto other generic HRQoL-tools to enable comparability across disease areas and healthcare systems. Given the differing recall periods of the IPOS and the EQ-5D-5L, meaningful mapping between those two measures is unlikely to be feasible with the current versions. The ICECAP-SCM, with its focus on compassionate care, could complement existing measures and provide a more holistic view of patient wellbeing. However, the ICECAP-SCM may not provide sufficient information on physical suffering to support patient-centered care and may not justify the additional patient burden [58]. Future research should focus on validating the ICECAP-SCM in different PC settings and cultural contexts.

### Strengths and limitations

Our study offers the first comprehensive psychometric validation of the ICECAP-SCM in specialist hospital inpatient PC settings, a population often underrepresented in research. It also makes the first psychometric evaluation of PC PROMs in the Austrian context and allows in-depth explorations of specific comparative symptom assessments between self-reports and clinician-reports.

Despite these strengths, the study has some limitations. First, the sample included a larger proportion of patients with lower educational levels compared to the general population, which may limit the representativeness and generalizability of the findings. Second, the study relies on self-reported data from patients with PC needs, introducing potential recruitment bias. Patients who felt better may have been more likely to participate, potentially leading to an overrepresentation of individuals in better health states. To mitigate this, participants who were unable to complete the questionnaire independently were offered assistance from healthcare professionals or relatives/friends. However, this support, which ranged from basic help to more involved, interview-like assessments, may have influenced the responses, and it is unclear to what extent this assistance affected the results. Third, this study used only paper-based PROMs due to limited feasibility of electronic data collection in the participating centers. This method may not fully reflect current data collection practices, can introduce data entry errors, and is more time-consuming. Finally, no Austrian value set is available for either the ICECAP-SCM or the EQ-5D-5L. Therefore, we used German value sets, which may not fully reflect cultural preferences in Austria.

## Conclusion

This study provides initial evidence of the construct validity, responsiveness and structural validity of the ICECAP-SCM capability-wellbeing measure in specialist PCUs in the Austrian hospital context. While the ICECAP-SCM may offer a more holistic view compared to the EQ-5D-5L, the observed ceiling effects may limit its applicability in specialist PCUs. This aspect needs to be considered together with other findings on the limited feasibility of PROM data collection in specialist PC [26, 58, 59] when planning PROM assessment in this context.

## Electronic supplementary material

Below is the link to the electronic supplementary material.

Supplementary Material 1

Supplementary Material 2
