# Supplementary material for: Psychometric properties of the ICECAP-SCM capability-wellbeing measure in specialist palliative care units in Austria
Source: Qual Life Res. 2025 Jul 23;34(10):2821–33. doi: 10.1007/s11136-025-04032-8 (PMC12535525; doi:10.1007/s11136-025-04032-8)
Supplement: Supplementary file 1 — Supplementary Material 1 [file 11136_2025_4032_MOESM1_ESM.docx]

**COSMIN Reporting guideline for studies on measurement properties of patient reported outcome measures**

Joel J Gagnier, Jianyu Lai, Lidwine B Mokkink, Caroline B Terwee. COSMIN reporting guideline for studies on measurement properties of patient-reported outcome measures. Qual Life Res. 2021 Aug; 30(8):2197-2218. doi: 10.1007/s11136-021-02822-4.

| **General Reporting recommendations relevant for all studies on measurement properties** | | | | |  |  |
| --- | --- | --- | --- | --- | --- | --- |
| **Item Number** | **Item Name** | **Item Description** | | **Page/Section** |  |  |
| **Report section: Title** | | | |  |  |  |
| T1 | Patient Reported Outcome Measure (PROM) | The name of the PROM instrument(s) (and version if relevant) being studied | | Title |  |  |
| T2 | Measurement Property (MP) | What MPs are being studied or more generally, that MPs are being studied (if there are many properties being investigated, for example) | | Title |  |  |
| T3 | Study sample | General description of relevant study sample characteristics (e.g., condition of interest, language) and also any intervention or exposure (e.g., treatments) if applicable. | | Title |  |  |
| **Report section: Abstract** | | | |  |  |  |
| A1 | PROM | The name of the PROM instrument(s) (and version if relevant) being studied (i.e. the SF-36 or SF-12; language version) or if it concerns an item bank (e.g., PROMIS instruments). The type of instrument (e.g. a self reported questionnaire or interview). | | Abstract-Purpose & Methods |  |  |
| A2 | Measurement Property | What MPs are being studied or more generally, that MPs are being studied (if there are many properties being investigated, for example) | | Abstract-Methods |  |  |
| A3 | Design | The type of study being used to test the properties (e.g., test-retest design, longitudinal study, cohort, cross sectional, case series, randomized etc.). Other details of the study design if relevant (intervention/exposure, description of comparison instruments, outcomes other than PROMs). | | Abstract-Methods |  |  |
| A4 | Sample | Inclusion / exclusion criteria. General description of relevant study sample characteristics (e.g., condition of interest, geographic location, language, other relevant demographic and baseline characteristics) | | Abstract-Methods |  |  |
| A5 | Methods | A brief description of the methods for investigating each MP including statistical analyses | | / |  |  |
| A6 | Results | The main results for all MPs investigated reporting statistics for each result with measures of precision where appropriate. | | Abstract-Results |  |  |
| A7 | Discussion/Conclusions | A brief description of the results in the context of existing evidence, main strengths and drawbacks and the need for future research on the PROM(s) investigated. | | Abstract-Conclusion |  |  |
| **Report section: Introduction** | | | |  |  |  |
| I1 | Name and describe the PROM of interest | Specify the name, type, language, and version of the PROM being investigated and how it was developed. Describe the construct the PROM aims to measure and its subscales; describe the structure of the PROM (e.g., the number of factors, the number of items, scoring algorithm); describe relevant instructions (like time period), and number or type of response categories. State whether the PROM is based on a reflective or formative model.  Note: This information may also appear in the methods section in greater detail. | | p.4, pp.6-7 |  |  |
| I2 | Target population | Describe the specific target population that the PROM was designed for. The authors need to provide the appropriate and necessary characteristics of this population. | | p.5 |  |  |
| I3 | Citation for the original development of the PROM | The citation for the original development paper(s) should be provided and other highly relevant citations related to the quality of the specific PROM under investigation. | | p.4 |  |  |
| I4 | State of Knowledge & Rationale | A description of the current scientific knowledge (what is known) regarding the MPs of? the PROM under investigation. The authors should provide a literature review or refer to a recent review of all existing evidence of the specific version (e.g., language, short form) of the PROM and explain why the new study is necessary and important. The rational for the current proposed study should be given. | | p.4 |  |  |
| I5 | Definitions | Specialized terms should be defined or explained. | | pp.3-4, pp.7-9 |  |  |
| I6 | Objectives and Hypotheses | State the specific objective(s) of the research and hypotheses related to the specific PROM under investigation. | | p.4, p.8 |  |  |
| **Report section: General Methods** | | | |  |  |  |
| GM1 | Study Design | State the key elements of the study design | | p.5 |  |  |
| GM2 | Participants | State how the participants were chosen; the inclusion and exclusion criteria. (e.g., if a PROM for a specific condition, then the eligibility and selection criteria should reflect this). | | p.5 |  |  |
| GM3 | PROM administration | An explicit description of how and when the PROM(s) were administered (e.g., in what setting) including data collection devices/system used (e.g. paper based, electronic administration / ePRO) should be provided. | | p.5 |  |  |
| GM4 | Data collection procedures | Provide information about other data collection, exposure methods (e.g., allocation to interventions) and time points / follow-up points. | | p.5 |  |  |
| GM5 | Power/sample size calculation | Provide a power calculation for all MP analyses. Alternatively, if a rule of thumb is used, state it and the source/citation. | | n/a |  |  |
| GM6 | Statistical analyses | Statistical analyses and tests corresponding to all hypotheses or objectives for all MPs should be reported. Where appropriate, a cut-off for statistical significance should be reported (e.g., p-value less than 0.05). A description of all statistics to be used to estimate the magnitude and direction of effect should also be reported, together with measures of variability or precision. Report statistical package used. | | pp. 7-10 |  |  |
| GM7 | Missing data | State approaches or plan for dealing with missing data. | | p.10 |  |  |
| GM8 | Post hoc analysis | The report should specify analyses that used data after the data collection period concluded (i.e., if the analyses were post hoc; secondary data analyses) and describe the rationale for any post hoc analyses. | | n/a |  |  |
| **Report section: General Results** | | | |  |  |  |
| GR1 | Missing data | The amount and reasons for missing data should be explained for all analyses for all PROMs (or other outcome measurement instruments) and relevant groups. | | p.11, Table 2 |  |  |
| GR2 | Participant/patient Characteristics | The study patients’ characteristics should be described, including baseline PROM scores. | | Table 2 |  |  |
| GR3 | Sample size | If one study contained analyses using different sample sizes, the authors should report the sample size for each analysis. | | Table 4, Table 5 |  |  |
| **Report section: Discussion** | | | |  |  |  |
| D1 | MP evidence | Per measurement property the authors should compare the result to the criteria for good measurement properties (e.g., COSMIN criteria) [27], and determine if the specific MP is sufficient or not. Note: This information may also appear in the results section in greater detail in a table for example. | | pp.21-23 |  |  |
| D2 | Practical relevance | The authors need to discuss the practical relevance of the findings. | | pp.23-24 |  |  |
| D3 | Strengths and limitations | Strengths and limitations of the study should be discussed. For example, discuss if there were any significant potential biases in the study that could have impacted the results. | | pp.24-25 |  |  |
| D4 | Generalizability | Generalizability issues related to the PROM results should be discussed. For example, discuss if the results could be generalized to other populations given the sample studied. | | pp.24-25 |  |  |
| D5 | Instrument changes | Discuss the need for modifications to the existing PROM or new PROM development. If you conclude that one of the measurement properties is insufficient, you could suggest some modification, or if it is really poor, you could suggest stopping use of the PROM (in the specific population or in general). | | pp.20-21 |  |  |
| D6 | Future Research | Report specifically the type of research needed to answer new questions arising out of these findings for the particular MP and PROM investigated. | | pp.23-24 |  |  |
| **Report section: Conclusions** | | |  | | |  |
| C1 | Conclusions | State the overall conclusions for each MP and of the use PROM investigated. | | p.25 | | |
| **Report section: Other information** | | |  | | |  |
| O1 | Conflict of Interest | State any relevant conflict of interest related to the PROM under investigation (e.g., an author being the PROM developer, funding body etc). | | n/a | | |
