# Supplementary material for: Psychometric properties of the ICECAP-SCM capability-wellbeing measure in specialist palliative care units in Austria
Source: Qual Life Res. 2025 Jul 23;34(10):2821–33. doi: 10.1007/s11136-025-04032-8 (PMC12535525; doi:10.1007/s11136-025-04032-8)
Supplement: Supplementary file 2 — Supplementary Material 2 [file 11136_2025_4032_MOESM2_ESM.docx]

**Appendix**

**A1. Known-groups validity – Categorization of groups**

The Karnofsky Performance Status is a scale ranging from 0 to 100, with higher scores indicating higher performance. The groups were formed according to the instructions in the measurement guidelines. Scores of 0-40 indicate low performance, 50-70 indicate moderate performance, and 80-100 indicate high performance [1]. The Numeric Rating Scale (NRS) pain score, which ranges from 0 to 10, was grouped into four categories following evidence on the optimal cut-off-point scheme for such scales [2]: "no pain" (score = 0), "mild pain" (score = 1 - 3), "moderate pain" (score = 4 - 6), and "severe pain" (score = 7 - 10). The IPOS symptom score, ranging from 0-40, was grouped into three categories: “Severe” (>20-40), “Moderate” (>10 to ≤20) and “Low” (≤10). This categorization is based on a sum score of symptoms affecting patients, with individual symptom scores ranging from 0 (not at all) to 4 (overwhelmingly). Scores below 10 indicate fewer or less severe symptoms, while scores above 20 indicate a high symptom burden.

**References**

[1] Schag, C. C., Heinrich, R. L., & Ganz, P. A. (1984). Karnofsky performance status revisited: reliability, validity, and guidelines. Journal of Clinical Oncology, 2(3), 187–193.

[2] Boonstra, A. M., Schiphorst Preuper, H. R., Balk, G. A., & Stewart, R. E. (2014). Cut-off points for mild, moderate, and severe pain on the visual analogue scale for pain in patients with chronic musculoskeletal pain. PAIN, 155(12), 2545–2550.

**A2. Distribution of ICECAP-SCM sum scores at baseline**

**
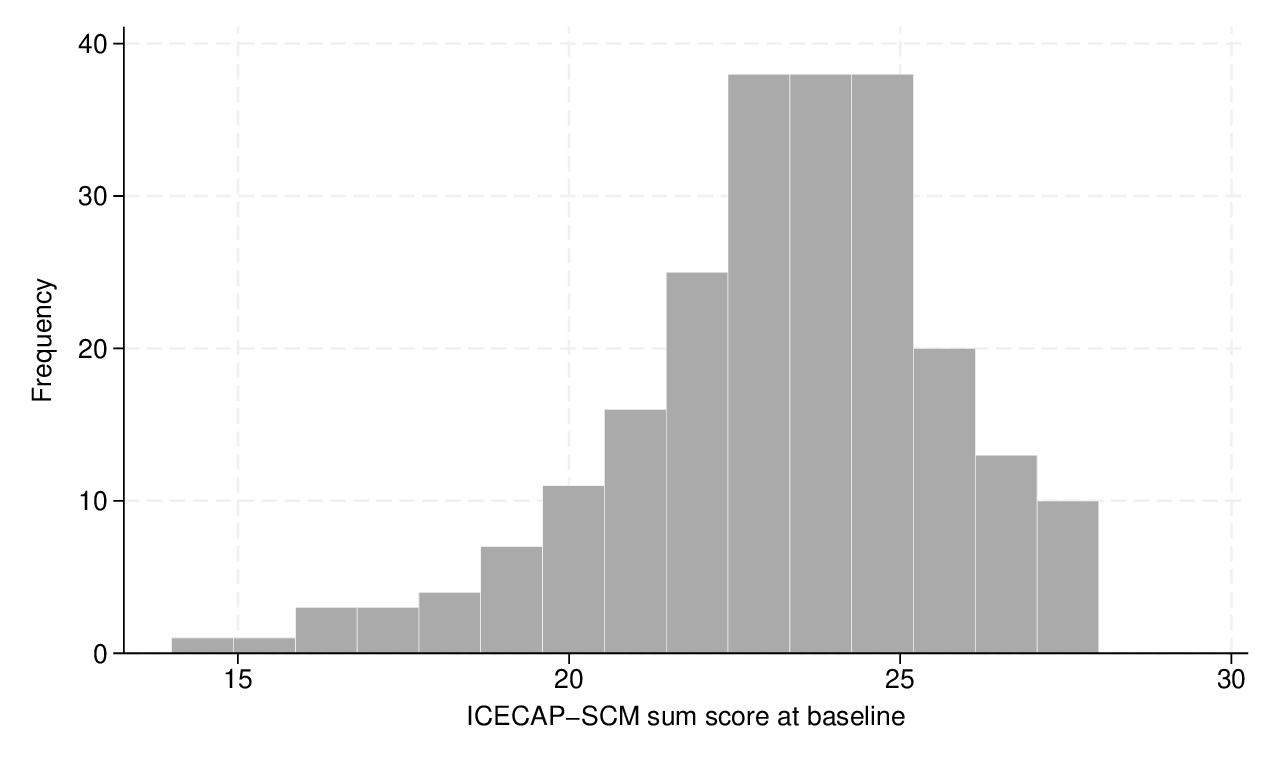
**

**Fig. 3** Histogram of ICECAP-SCM sum scores (7-28) at baseline (n=228)

**A3. Responsiveness**

**Scheffe Post-hoc tests as supplement to the one-way ANOVA (Table 7)**

**Table 7** Scheffe Post-hoc tests for one-way ANOVA for responsiveness analyses

| ICECAP-SCM | Worsened vs. No change | Improved vs. No change | Improved vs. Worsened |
| --- | --- | --- | --- |
| Clinician’s assessment | 0.287 | 0.770 | 0.069 |
| EQ-5D-5L | 0.131 | 0.414 | 0.014* |
| EQ-VAS | 0.495 | 0.765 | 0.254 |
| IPOS | 0.142 | 0.039* | 0.002* |

Note: EQ-VAS=Visual analogue scale, ICECAP-SCM = ICECAP-Supportive Care Measure, IPOS = Integrated Palliative care Outcome Scale

**Agreement in change between ICECAP-SCM scores and anchor instruments**

Among the patients who showed improvements in the ICECAP-SCM score, different proportions improved according to the clinician assessment (31%), EQ-5D-5L (44%), EQ-VAS (35%), and IPOS (43%) (Table 8). Of those who did not change, 46% (clinician assessment), 60% (EQ-5D-5L), 52% (EQ-VAS) and 64% (IPOS) did not change in the ICECAP-SCM score. For the worsened groups it was 22% (clinician assessment), 25% (EQ-5D-5L), 16% (EQ-VAS) and 50% (IPOS), that had also worsened according to the ICECAP-SCM.

**Table 8** Number of patients improved, deteriorated or unchanged as per the ICECAP-SCM score compared to other anchor instruments

| Instrument  (no. of complete cases) | Change in instrument scores |  | Change in ICECAP-SCM score | | |
| --- | --- | --- | --- | --- | --- |
|  |  | n | Improved | Worsened | No change |
| Clinician's assessment | Improved | 80 | **25 (31%)** | 10 (13%) | 45 (56%) |
| (n=144) | Worsened | 23 | 4 (17%) | **5 (22%)** | 14 (61%) |
|  | No change | 41 | 16 (39%) | 6 (15%) | **19 (46%)** |
| EQ-5D-5L | Improved | 50 | **22 (44%)** | 5 (10%) | 23 (46%) |
| (n= 148) | Worsened | 28 | 7 (25%) | **7 (25%)** | 14 (50%) |
|  | No change | 70 | 19 (27%) | 9 (13%) | **42 (60%)** |
| EQ-VAS | Improved | 48 | **17 (35%)** | 6 (13%) | 25 (52%) |
| (n=148) | Worsened | 25 | 6 (24%) | **4 (16%)** | 15 (60%) |
|  | No change | 75 | 25 (33%) | 11 15%) | **39 (52%)** |
| IPOS | Improved | 74 | **32 (43%)** | 4 (5%) | 38 (51%) |
| (n=148) | Worsened | 16 | 4 (25%) | **8 (50%)** | 4 (25%) |
|  | No change | 58 | 12 (21%) | 9 (16%) | **37 (64%)** |

Note: EQ-VAS=Visual analogue scale, ICECAP-SCM = ICECAP-Supportive Care Measure, IPOS = Integrated Palliative care Outcome Scale; The categorization of the changes in scores between baseline and 1-week follow-up in improved, worsened or no change was based on a change of 0.5 standard deviation of the mean baseline score except for the clinician’s assessment. Bold indicates values in agreement.

**Construct approach to assess responsiveness**

To assess responsiveness, we additionally employed the construct approach as an alternative to the anchor-based approach. The construct approach, similar to the approach used for assessing convergent validity, consists of testing hypotheses about expected correlations between changes in scores on the instrument of interest and changes in scores on other instruments known to have adequate responsiveness. We tested pre-defined hypotheses regarding the expected correlations between the change scores of the PROMs (ICECAP-SCM, EQ-5D-5L, IPOS and EQ-VAS) with Spearman rank correlations, as presented in Table 9 and Table 10.

**Table 9** Hypotheses about correlations between change scores of ICECAP-SCM, EQ-5D-5L, IPOS and EQ-VAS

| Hypotheses | | Met |
| --- | --- | --- |
| 1 | The ICECAP-SCM change scores are expected to correlate on at least moderate level (r ≥ 0.3 or r ≤ -0.30) with EQ-5D-5L and IPOS. | N |
| 2 | The ICECAP-SCM change scores are expected to show a weak correlation (r ≤ 0.3) with EQ-VAS as these are two different constructs. | Y |
| 3 | The correlation between EQ-5D-5L and EQ-VAS change scores is expected to be at least moderate (r ≥ 0.3). | N |
| 4 | The correlation between IPOS and EQ-5D-5L change scores is expected to be at least moderate (r ≥ 0.3). | Y |

Note: EQ-VAS=Visual analogue scale, ICECAP-SCM = ICECAP-Supportive Care Measure, IPOS = Integrated Palliative care Outcome Scale, N = No, Y = Yes;

**Table 10** Spearman rank correlations of change scores between baseline and follow-up 1 assessments (n=148)

|  | ICECAP-SCM | EQ-5D-5L | IPOS | EQ-VAS |
| --- | --- | --- | --- | --- |
| ICECAP-SCM | 1 | 0.29** | -0.29** | 0.09 |
| EQ-5D-5L |  | 1 | *-0.40*** | 0.21* |
| IPOS |  |  | 1 | -0.26* |
| EQ-VAS |  |  |  | 1 |

Note: EQ-VAS=Visual analogue scale, ICECAP-SCM = ICECAP-Supportive Care Measure, IPOS = Integrated Palliative care Outcome Scale; ** p < 0.001, * p < 0.05; bold indicates strong correlation, italic indicates moderate correlation; ICECAP-SCM and EQ-5D-5L refer to the preference-based values and IPOS to the sum scores.

**A4. Exploratory factor analysis – Scree plot of eigenvalues**

**
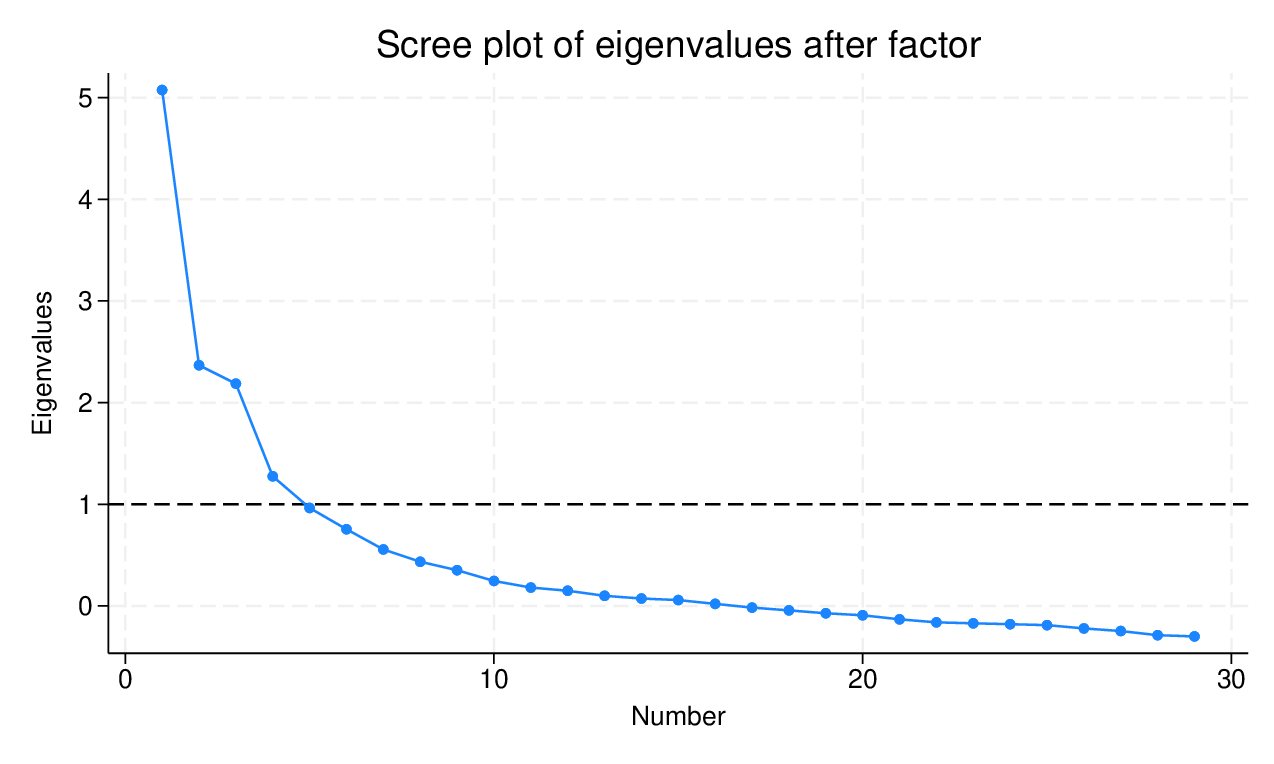
**

**Fig. 4** Scree plot of eigenvalues (all items)
